# Psychometric assessment of the Beck anxiety inventory and key anxiety determinants among Ukrainian female refugees in the Czech Republic

**DOI:** 10.3389/fpsyg.2024.1529718

**Published:** 2025-01-15

**Authors:** Iryna Mazhak, Danylo Sudyn

**Affiliations:** ^1^School of Population Health, RCSI University of Medicine and Health Sciences, Dublin, Ireland; ^2^Department of Sociology, National University of Kyiv-Mohyla Academy, Kyiv, Ukraine; ^3^Department of Sociology, Ukrainian Catholic University, Lviv, Ukraine

**Keywords:** anxiety, self-reported physical and mental health, female refugees, Ukraine, Beck anxiety inventory

## Abstract

**Introduction:**

The full-scale Russian war has caused Ukrainian female refugees to experience many stressful events which may have an adverse impact on their mental health. Understanding the prevalence and determinants associated with anxiety is essential for psychosocial support. The study aimed: to evaluate the psychometric validity of the Ukrainian version of the Beck Anxiety Inventory (BAI) among Ukrainian female refugees in the Czech Republic, to determine the prevalence of anxiety, and to identify key determinants for anxiety in this population.

**Methods:**

Anxiety was measured by BAI, which was validated by applying confirmatory factor analysis. Linear regressions were run to understand associations between social, physical and mental health determinants and anxiety, adjusted by socio-demographics.

**Results:**

The BAI had a high level of internal consistency. External consistency was confirmed through: structural validity via CFA, indicating that a four-factor model, including cognitive, autonomic, neuromotor, and panic factors, were the most appropriate for the Ukrainian version of BAI; and convergent validity, shown by significant correlations between the total scores of the BAI and coping strategies, perceived stress, depression as well as self-reported physical and mental health. The study revealed that more than half of the participants had moderate to concerning symptoms of anxiety. The analysis indicated that poor perceived health, ineffective coping strategies, high perceived stress, and hampered daily activities due to health issues, are significant predictors of increased anxiety. Conversely, positive or stable social relations with relatives, neighbors, and locals, and the absence of discrimination, were shown to be crucial in reducing anxiety levels.

## 1 Introduction

The Czech Republic is one of the countries that has offered “temporary protection” to Ukrainian refugees since Russia’s full-scale invasion of Ukraine in 2022. These Ukrainian refugees, especially females with children under 18, deal with numerous stressful events, which might lead to an increase in mental health problems, including anxiety. In refugee and forced migrant populations, anxiety is commonly understood to be one of the most common psychological problems. In addition, there are reinforcing factors such as trauma, uncertainty, and the difficulties of resettlement.

Among psychological tools to measure anxiety, the Beck Anxiety Inventory (BAI) is one of the most popular and well-established self-report instruments designed to evaluate the severity of anxiety symptoms across diverse populations. It was developed by Beck et al. (1988) and Beck and Steer (1991, 1993) as a 21-item self-report questionnaire. Although the BAI is widely used in different languages and populations, little is known about its psychometric qualities in the context of Ukrainian refugees and the Ukrainian version of the tool. In order to check the BAI’s validity and cultural sensitivity, this study aims to validate its psychometric qualities among Ukrainian female refugees in the Czech Republic.

### 1.1 Psychometric validation of BAI

The psychometric validity of the BAI, including its internal consistency and structural and convergent validity, has been validated across different general and clinical populations and across diverse cultural and language contexts.

Firstly, the BAI’s factor structure and psychometric qualities have been examined in different samples with various mental health conditions. Beck et al. applied promax rotation and iterated principal factor axis analysis and revealed a two-component structure. The somatic factor included 12 items: numbness or tingling, feeling hot, wobbliness in legs, dizzy or lightheaded, heart pounding or racing, unsteady, hands trembling, shaky, scared, faint, face flushed, and sweating. The nine items that made up the cognitive factor were: unable to relax, fear of the worst happening, terrified, nervous, feelings of choking, fear of losing control, difficulty breathing, fear of dying, and indigestion (Beck et al., 1988). Two-factor structure, including cognitive and somatic symptoms, was also revealed in another study, in a sample of psychiatric patients. Notably, although males and females had similar factor structures, females scored higher on both cognitive and somatic symptoms and overall anxiety levels (Hewitt and Norton, 1993).

Secondly, through exploratory principal-component analyses using varimax rotation, a four-factor model was developed in the community adult sample (Osman et al., 1993). In Wetherell and Areán (1997) study with older medical outpatients where confirmatory factor analysis was applied, it supported a four-factor model of anxiety symptoms, including cognitive, autonomic, neuromotor, and panic symptoms. The cognitive factor included seven items: unable to relax; fear of the worst happening; terrified or afraid; nervous; fear of losing control; fear of dying; and scared. The autonomic factor included four items: feeling hot; indigestion; face flushing; and hot/cold sweats. The neuromotor factor included seven items: numbness or tingling; wobbliness in legs; dizzy or lightheaded; unsteady; shaky/unsteady; hands trembling; and faint/lightheaded. The panic factor included three items: heart pounding/racing; feeling of choking; and difficulty breathing (Wetherell and Areán, 1997). The BAI reported to have strong psychometric properties, including high internal consistency and structural and convergent validity, validated across diverse populations.

### 1.2 Anxiety risk factors among refugees

A growing body of studies underscores the high prevalence of anxiety among refugee and forced migrant populations across various contexts. For example, a study among Somali refugees in Kenya reported that 44.0% of participants had anxiety symptoms (Im et al., 2022). Among Iraqi refugees in Jordan, 60.8% exhibited high anxiety levels (Al-Smadi et al., 2017), and 27.5% of Syrian refugees in Ireland were also found to suffer from anxiety (Collins et al., 2022). Furthermore, 68% of undocumented adult migrants in Sweden experienced moderate to severe anxiety (Andersson et al., 2018); and a prevalence of anxiety symptoms was evidenced in 39.1% among migrants in Morocco (Essayagh et al., 2023). A systematic review by Henkelmann et al. (2020) reported anxiety prevalence rates among refugees resettling in high-income countries ranging from 13 to 42%. Similarly, a meta-analysis of adult Syrian refugees in Western countries by Nguyen et al. (2022) found a 40% prevalence rate. Moreover, another systematic review and meta-analysis of refugees and asylum seekers The Middle East and North Africa reported an anxiety prevalence of 43% (95% CI: 31, 57%) (Zabarra et al., 2024). Additionally, anxiety prevalence was 21% among labor migrants vs. 40% among refugees globaly (Lindert et al., 2009). These findings from various studies highlight the concerning prevalence of anxiety among refugee populations, though the reported rates differ significantly. Additionally, a study with refugees showed that 5 years after resettlement, the level of anxiety disorders increased (Giacco et al., 2018).

Several previous studies have identified common risk factors for anxiety among refugees. Such post-migration stressors as difficulties accessing healthcare, language barriers, unemployment, and social isolation, are thought to exacerbate anxiety. For example, according to Giacco et al. (2018), a connection was found between different aspects of poor integration, such as social isolation, unemployment and acculturation problems in the host country, and higher rates of mental disorders, including anxiety. In Morocco, the prevalence of anxiety symptoms among migrants was 39.1%, with factors such as diabetes, refugee status, overcrowding, stress, and low income being significant contributors (Essayagh et al., 2023). Additionally, accessing healthcare is often a challenge for refugees because of difficulties with healthcare system navigation, inadequate local language proficiency, and different approaches to understanding and treating mental disorders (Giacco et al., 2018). The research demonstrates that refugees face a multitude of risk factors that significantly contribute to the high prevalence of anxiety.

On the one hand, Ukrainian females, within the country and abroad as refugees, are increasingly asserting their autonomy and carving out new roles in response to the war. They are expanding their participation across various spheres of life by taking on new responsibilities, advocating for their rights, and challenging traditional gender norms (Phillips and Martsenyuk, 2023). On the other, as forced migrants, they face additional risks due to the challenges of the ongoing war, separation from family members, particularly male relatives engaged in combat, the pressure of adjusting to a new cultural environment, and uncertainties regarding legal status in host countries–these can further intensify anxiety. Additionally, difficulties with social integration in the Czech Republic may exacerbate mental health struggles during resettlement.

Among Ukrainians, anxiety levels were high even before displacement, with moderate and severe anxiety prevalent in 25.6 and 19.0% of non-displaced individuals, 25.7 and 23.4% of internally displaced individuals, and 26.2 and 25.8% of refugees (Lushchak et al., 2024). Research on refugee populations indicates that female refugees generally report higher anxiety levels than their male counterparts. This trend is attributed to increased exposure to gender-based violence, caregiving responsibilities for children and elders, and greater vulnerabilities during migration. For instance, Ukrainian female refugees in Germany reported significantly higher levels of mental health conditions compared to males: severe psychological distress (46.4% of females and 20% males), moderate to severe depression and anxiety (45% of females and 26% males) (Buchcik et al., 2023).

This study aims to (1) evaluate the psychometric validity and reliability of the Ukrainian version of the BAI among Ukrainian female refugees in the Czech Republic, (2) determine the prevalence of anxiety, and (3) identify key determinants for anxiety in this population. By doing so, it seeks to contribute to the development of evidence-based mental health interventions tailored to the unique needs of Ukrainian female refugees.

## 2 Methodology

### 2.1 Participants

This cross-sectional study involved 919 Ukrainian female refugees residing in the Czech Republic. The eligibility criteria for participants were following: (1) be 18 years of age or older, and (2) have been forcibly displaced to the Czech Republic as a result of the full-scale Russian invasion of Ukraine in 2022. Data were collected through an online survey administered in the Ukrainian language, which covered various aspects of self-reported physical health and psycho-emotional status, some mental health conditions, and socio-demographic characteristics.

The survey was disseminated through social media platforms used by Ukrainian refugees in the Czech Republic, such as Facebook, Telegram, and Viber. Additionally, non-governmental organizations that provide support to refugees and Czech schools that enroll Ukrainian children assisted in distributing the online survey.

All received responses were anonymized to ensure privacy and were handled by the General Data Protection Regulation. Before proceeding with the survey, participants were required to e-sign an online informed consent form, acknowledging that their participation was voluntary and that they could withdraw at any time without consequence. Data collection took place between June 6 and September 6, 2022.

### 2.2 Measures

#### 2.2.1 Online survey questionnaire

The online survey included several key components. Firstly, it included a socio-demographic component that gathered general information about the participants, such as their age, education level, socio-economic status, marital status, number of children under 18, and employment status. Secondly, the survey explored: self-reported physical health; participants’ access to healthcare; any health deterioration due to the war; the presence of chronic diseases; vaccination against COVID-19; registration with family doctors; and the impact of health issues on their daily activities. Thirdly, the survey assessed social health by inquiring about: participants’ relationships with family, friends, and locals; their participation in events organized for refugees; involvement in Ukrainian religious communities; cultural differences; and experiences of discrimination. Finally, the survey examined: coping strategies; perceived stress; self-reported psycho-emotional status; and access to psychological aid.

#### 2.2.2 Anxiety symptoms measurement

Anxiety symptoms were evaluated using the BAI, a self-reported instrument designed to assess both the frequency and severity of anxiety symptoms (Beck et al., 1993). Each item on the BAI is scored on a 4-point scale, ranging from 0 (not at all) to 3 (severely, I could barely handle it), with total scores spanning from 0 to 63. This scale allows for a comprehensive evaluation of anxiety, capturing a wide range of symptom severity levels.

The original English version of the BAI was translated into Ukrainian and then back into English by two independent translation agencies. This back-translation process was followed by a review conducted by a researcher and a psychologist to ensure that the translated version maintained both cultural and contextual relevance. The Ukrainian version of the BAI was then administered to participants, and the full translation is available in Appendix 1.

#### 2.2.3 Other mental condition measurements

Depression was measured by applying the Patient Health Questionnaire (PHQ-9). The PHQ-9 was developed as a screener for depression (PHQ-9; Kroenke et al., 2001). Additionally, perceived stress and coping strategies were also included in the analysis. Perceived stress was measured by the Perceived Stress Scale (PSS-14), which was invented by Cohen et al. (1983). PSS-14 measures how people assess the degree of control they have or do not have over unpredictable, unmanageable and overburdened events in their lives (Cohen and Williamson, 1988). The BRIEF-COPE inventory (Carver et al., 1989) was employed to investigate coping strategies for stressful events.

## 3 Results

Jamovi statistic software version 2.4.12.0 was utilized to perform the confirmatory factor analysis and in the BAI validation process and linear regression analysis (The jamovi project, 2023; R Core Team, 2022; Revelle, 2023; Fox and Weisberg, 2020; Gallucci and Jentschke, 2021; Rosseel, 2019; Epskamp et al., 2019).

### 3.1 Participant characteristics

The survey received 919 responses from Ukrainian female refugees. Over 70% of respondents reported being married or cohabiting, while 68.4% had children under 18. Only 30% were employed in the Czech Republic, with 76% indicating that their financial and material conditions had worsened since relocating. A significant portion, 75%, held a university degree. The two most significant challenges these refugees faced in adapting to life in the Czech Republic were financial difficulties, reported by 40% of respondents, and a lack of Czech language proficiency, which was cited by 81%.

Healthcare access was also a concern; only 28% of respondents had registered with a family physician. Regarding their self-reported physical health, 43% described their health as good, 46.8% as fair, and 10.2% as either bad or very bad. Additionally, 4.5% of participants reported that the war had injured or damaged their health, and 27.9% mentioned a deterioration in their health over the past month.

In terms of emotional and psychological well-being, 52.7% of female refugees rated their condition as fair, 26% as bad, and 7.7% as very bad. Most respondents employed effective coping strategies, with 64.5% using problem-focused coping strategies and 29.5% relying on emotion-focused coping. Most participants experienced moderate to high levels of perceived stress.

The responses from the female refugees were analyzed using descriptive statistics, focusing on absolute and relative frequencies, which are presented in Appendix 2 (Socio-demographic, self-reported physical and mental health statuses, anxiety, depression, coping strategies, and perceived stress characteristics).

### 3.2 BAI psychometric validation

It was revealed that more than half of the participants had moderate (31.3%) to concerning (22.1%) symptoms of anxiety. Moreover, the analysis of the data reveals a notable prevalence of severe anxiety-related symptoms among Ukrainian female refugees. The items “unable to relax” and “fear of the worst happening” stand out as particularly critical, with 37.6 and 42.3% of respondents, respectively, reporting these as severe. These high percentages, coupled with their high mean severity. Additionally, items such as “nervous” and “terrified or afraid” showed a significant percentage of severe responses (31.7 and 26.1%, respectively). The findings also indicate that symptoms like “heart pounding/racing” and “feeling unsteady” have substantial distributions across moderate to severe categories, suggesting a widespread impact on physical well-being (Table 1).

**Table 1:** Descriptive statistics, including means, standard deviation, and percentages of BAI (*N* = 919).

Item		Mean	SD	Response *n* (%)			
				Not at all	Mildly	Moderately	Severely
Q1	Numbness or tingling	0.92	0.97	375 (43.1)	260 (29.9)	165 (19.0)	70 (8.0)
Q2	Feeling hot	1.00	0.99	336 (38.8)	277 (31.9)	170 (19.6)	84 (9.7)
Q3	Wobbliness in legs	0.78	0.94	433 (50.5)	236 (27.5)	131 (15.3)	58 (6.8)
Q4	Unable to relax	1.93	0.99	72 (8.3)	246 (28.3)	224 (25.8)	327 (37.6)
Q5	Fear of worst happening	1.96	1.06	109 (12.5)	184 (21.1)	211 (24.2)	369 (42.3)
Q6	Dizzy or lightheaded	1.23	1.03	255 (29.6)	276 (32.0)	206 (23.9)	125 (14.5)
Q7	Heart pounding/racing	1.54	1.01	157 (18.0)	269 (30.8)	268 (30.7)	180 (20.6)
Q8	Unsteady	1.48	1.06	184 (21.5)	266 (31.1)	215 (25.2)	189 (22.1)
Q9	Terrified or afraid	1.61	1.05	145 (16.6)	278 (31.9)	221 (25.3)	228 (26.1)
Q10	Nervous	1.86	0.96	69 (7.9)	260 (29.8)	267 (30.6)	277 (31.7)
Q11	Feeling of choking	0.90	1.01	402 (47.0)	223 (26.1)	147 (17.2)	83 (9.7)
Q12	Hands trembling	0.94	1.00	377 (43.6)	253 (29.3)	147 (17.0)	87 (10.1)
Q13	Shaky/unsteady	0.64	0.89	498 (58.5)	211 (24.8)	92 (10.8)	50 (5.9)
Q14	Fear of losing control	1.22	1.10	287 (33.6)	249 (29.1)	160 (18.7)	159 (18.6)
Q15	Difficulty in breathing	0.69	0.95	499 (58.0)	199 (23.1)	96 (11.1)	67 (7.8)
Q16	Fear of dying	0.96	1.11	415 (48.3)	198 (23.1)	116 (13.5)	130 (15.1)
Q17	Scared	1.14	1.09	312 (36.7)	254 (29.8)	140 (16.5)	145 (17.0)
Q18	Indigestion	1.03	1.07	354 (41.5)	246 (28.9)	127 (14.9)	125 (14.7)
Q19	Faint/lightheaded	0.52	0.83	556 (65.4)	185 (21.8)	69 (8.1)	40 (4.7)
Q20	Face flushed	0.84	0.99	424 (49.6)	225 (26.3)	129 (15.1)	77 (9.0)
Q21	Hot/cold sweats	1.17	1.09	297 (34.5)	263 (30.6)	(7.8)	147 (7.1)

### 3.3 Reliability (internal consistency of the BAI)

The BAI is widely recognized for its robust use in assessing anxiety severity. Reliability (internal consistency) of the BAI was measured with Cronbach’s *α* (acceptability criterion of which is ≥0.70) and McDonald’s *ω* (acceptability criterion of which is ≥0.65).

In this study, we examine the internal consistency of the BAI total scale and its subscales as defined by different factor models: one-factor, the two-factor models proposed by Beck et al. (1993) and Hewitt and Norton (1993), and the four-factor model by Wetherell and Areán (1997) (Appendix 3).

Regarding factor models, Beck et al.'s (1993) two-factor model categorizes the BAI into ‘Somatic’ and ‘Cognitive’ subscales. The ‘Somatic’ subscale, comprising 12 items (1, 2, 3, 6, 7, 8, 12, 13, 17, 19, 20, 21), focuses on physical symptoms of anxiety and demonstrates excellent internal consistency with a Cronbach’s *α* of 0.89/McDonald’s *ω* of 0.89. The ‘Cognitive’ subscale, which includes nine items (4, 5, 9, 10, 11, 14, 15, 16, 18), captures the mental and emotional components of anxiety and has a Cronbach’s α of 0.84/McDonald’s ω of 0.85, indicating good internal consistency.

In contrast, Hewitt and Norton's (1993) two-factor model extends the ‘Somatic’ subscale to 14 items (1, 2, 3, 6, 7, 8, 11, 12, 13, 15, 18, 19, 20, 21) and yields an even higher internal consistency with a Cronbach’s *α* of 0.90/McDonald’s *ω* of 0.91. This enhancement suggests a more robust measure of somatic symptoms when including additional items. The ‘Cognitive’ subscale, consisting of seven items (4, 5, 9, 10, 14, 16, 17), maintains good internal consistency with a Cronbach’s *α* of 0.85/McDonald’s *ω* of 0.85, supporting the effectiveness of this model.

The four-factor model of Wetherell and Areán (1997) offers ‘Cognitive,’ ‘Autonomic,’ ‘Neuromotor,’ and ‘Panic’ subscales. The ‘Cognitive’ subscale, retaining the same seven items as in the previous models, shows a Cronbach’s *α* of 0.85/McDonald’s *ω* of 0.85, underscoring its reliability in measuring cognitive anxiety. The ‘Autonomic’ subscale, comprising four items (2, 18, 20, 21), measures autonomic nervous system responses to anxiety and has a Cronbach’s α of 0.73/McDonald’s ω of 0.74, indicating acceptable internal consistency. The ‘Neuromotor’ subscale, with seven items (1, 3, 6, 8, 12, 13, 19), focuses on neuromotor symptoms and demonstrates good internal consistency with a Cronbach’s α of 0.83/McDonald’s ω of 0.84. Finally, the ‘Panic’ subscale, including three items (7, 11, 15), targets panic-related symptoms and has a Cronbach’s α of 0.77/McDonald’s ω of 0.79 and suggests these items are reliable in capturing panic symptoms specifically.

The results indicate that both Cronbach’s α and McDonald’s ω exceeded commonly accepted thresholds. Although the one-factor model demonstrated the highest reliability, both two-factor models also displayed excellent reliability. The four-factor model showed acceptable to excellent reliability, albeit with increased complexity in interpreting the various dimensions of anxiety. Overall, the BAI’s reliability remains robust across models, making it a highly reliable tool for assessing anxiety severity.

### 3.4 Validity of the BAI

#### 3.4.1 Structural validity

Structural validity was assessed by confirmatory factor analysis (CFA). Model fit was checked by the comparative fit index (CFI), Tucker-Lewis’s index (TLI), standardized root mean square residual (SRMR), and root mean square error of approximation (RMSEA). The acceptable fit criteria were CFI ≥ 0.95, TLI ≥ 0.95, SRMR ≤0.06, and RMSEA ≤0.06. A confirmatory approach was applied by testing the one-, two-, and four-factor models (Appendix 4) and Table 2 presents a comparison between them.

**Table 2:** Confirmatory factor analysis was performed for two- and four-factor models, and bifactor model analysis for the four-factor model.

Factor Model	Items	CFI	TLI	SRMR	RMSEA
One-factor	1–21	0.967	0.963	0.084	0.100
Two-factor (Beck et al.’s model)	Somatic (1, 2, 3, 6, 7, 8, 12, 13, 17, 19, 20, 21)	0.969	0.965	0.083	0.098
	Cognitive (4, 5, 9, 10, 11, 14, 15, 16, 18)				
Two-factor (Hewitt and Norton’s model)	Somatic (1, 2, 3, 6, 7, 8, 11, 12, 13, 15, 18, 19, 20, 21)	0.982	0.980	0.065	0.074
	Cognitive (4, 5, 9, 10, 14, 16, 17)				
Four-factor (Wetherell and Areán)	Cognitive (4, 5, 9, 10, 14, 16, 17)	0.985	0.982	0.062	0.069
	Autonomic (2, 18, 20, 21)				
	Neuromotor (1, 3, 6, 8, 12, 13,19)				
	Panic (7, 11, 15)				
The bifactor model (based on Wetherell and Areán)	Cognitive (4, 5, 9, 10, 14, 16, 17)	0.985	0.983	0.062	0.069
	Autonomic (2, 18, 20, 21)				
	Neuromotor (1, 3, 6, 8, 12, 13,19)				
	Panic (7, 11, 15)				
	General (1–21)				

The analysis demonstrated that the one-factor model, which includes all 21 items, shows a relatively high CFI (0.967) and TLI (0.963), suggesting a reasonable fit. However, the SRMR (0.084) and the RMSEA (0.100) indicate a poor fit. Then, the two-factor model (Beck et al., 1993) that divides the items into somatic and cognitive factors, showed a slight improvement in fit compared to the one-factor model, with a bit higher CFI (0.969) and TLI (0.965) and a somewhat lower SRMR (0.083). However, the RMSEA (0.098) remains problematic. Hewitt and Norton's (1993) two-factor model also divides the items into somatic and cognitive factors but with a different item grouping. This model shows a significant improvement in fit, with a CFI of 0.982, TLI of 0.980, SRMR of 0.065, and RMSEA of 0.074. These indices indicate a much better fit than the previous models, suggesting that the specific item groupings in this two-factor structure better represent the data. The four-factor model proposed by Wetherell and Areán (1997) divides the items into cognitive, autonomic, neuromotor, and panic factors. This model provides the best overall fit, with a CFI of 0.985, TLI of 0.982, SRMR of 0.062, and RMSEA of 0.069. These indices are well within acceptable ranges, indicating that the four-factor model captures the underlying structure of the data more accurately than the previous models. The bifactor model, which adds a general factor alongside the four specific factors, shows nearly identical fit indices to the four-factor model, with a CFI of 0.985, TLI of 0.983, SRMR of 0.062, and RMSEA of 0.069. Therefore, the four-factor model offers the best fit for the data. The bifactor model does not substantially improve upon the four-factor model, suggesting that the general factor is not necessary to explain the variance.

**Figure 1:**
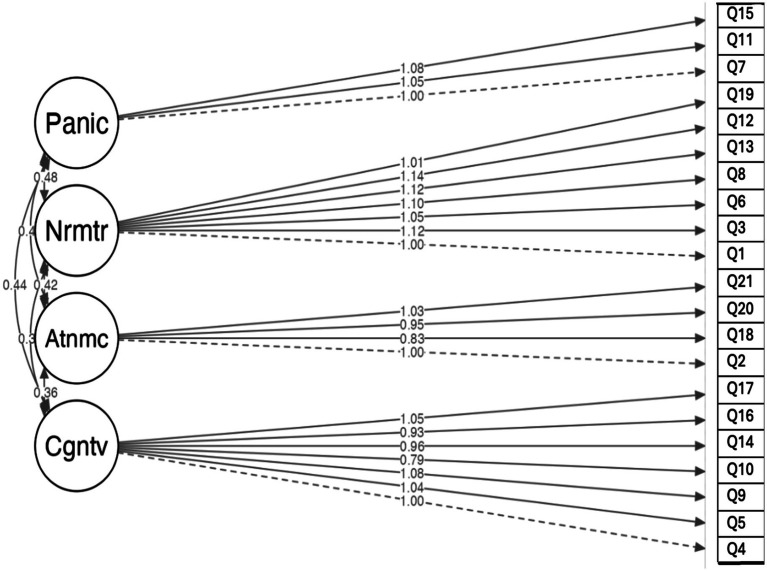
Structure of the BAI for Ukrainian female refugees in the Czech Republic based on the confirmatory factor analysis.

This model includes all 21 items distributed across four specific factors, providing a robust framework for understanding the underlying structure (Figure 1).

#### 3.4.2 Convergent validity

Convergent validity was assessed by correlating the total scores of the BAI and the BRIEF-COPE (three-factor structure: problem-focused, emotional-focused and avoidant strategies), Self-Reported Physical Health (SRPH) and Self-Reported Emotional and Psychological Status (SREPS), perceived stress (PSS-14) and depression (PHQ-9). Pearson’s correlation coefficients of |0.10| were considered as small of |0.30| as moderate, and of |0.50| or higher as strong correlations (Appendix 5–The Correlation Matrix). All correlations were significant at levels of 0.001.

Correlations between the Ukrainian version of BAI and related measurements of mental health conditions provided evidence for the concurrent validity of the BAI. The correlation between BAI total scores and depression levels, as measured by the PHQ-9, was found to be r = 0.69. This strong correlation suggests a significant overlap in the symptoms of anxiety and depression, supporting that anxiety coexists with depression. Similarly, the correlation between the BAI total score and perceived stress levels was *r* = 0.60, indicating a strong positive relationship. This underscores the role of stress in the experience of anxiety, showing that higher perceived stress is associated with increased anxiety levels. Furthermore, the BAI total score showed a strong correlation of r = 0.57 with measures of self-reported emotional and psychological status. This suggests that individuals who report higher levels of anxiety also tend to report poorer emotional and psychological well-being. In examining coping strategies, the relationship between the BAI total score and the use of avoidant coping strategies was *r* = 0.53, indicating a strong correlation. This suggests that individuals with higher anxiety levels are more likely to engage in avoidance behaviors as a coping strategy. Lastly, the correlation between the BAI total score and self-reported physical health was moderate (*r* = 0.39), this implies that higher anxiety may be associated with poorer perceived physical health, albeit to a lesser extent than its association with self-reported emotional and psychological status.

### 3.5 Anxiety and its key determinants

Linear regression analysis was performed to investigate the associations between anxiety and some physical health factors, mental health measurements and social risk factors. Linear regressions (Appendix 6–Linear Regressions Outcomes) were run to understand associations of anxiety with physical health determinants (Model 1), social determinants (Model 2), and mental health determinants (Model 3). The Final Model included all significant determinants, with depression as covariant. All models were adjusted by the socio-demographics of the sample. All statistical analyses were performed at a 5% level of significance. The scatterplots generated to assess linearity indicated linear relationships. All Models were statistically significant.

#### 3.5.1 Physical health determinants

Model 1 established that factors related to physical health determinants used in regression could statistically significantly predict the level of anxiety and accounted for 27.5% of the explained variance in anxiety measured by BAI. Linear regression analysis showed that females whose physical health remained unchanged reported lower levels of anxiety (*β* = −2.79) compared to those whose situation with health worsened during the last month. Participants who reported health deterioration due to the war (*β* = 6.81) had significantly higher anxiety scores compared to others. Similarly, females who reported a need for continuous medical supervision had higher anxiety scores (*β* = 2.66) compared to those who did not need it. There was a strong relationship between decreasing self-reported physical health from very good to good (*β* = 4.97), fair (*β* = 8.49), bad (*β* = 13.73), and very bad (*β* = 17.16) with increasing anxiety score. Additionally, respondents who reported their daily activities being hampered due to health issues (*β* = 3.76) have significantly higher anxiety scores compared to those who did not. There are also associations between COVID-19 vaccination and anxiety, therefore, females who have received two doses (*β* = 2.37) and three doses (*β* = 3.78) have significantly higher anxiety scores compared to those who have not received any doses. The analysis shows that for each unit increase in age groups, the anxiety decreases by approximately 1.57 units. Moreover, respondents who report that their economic status did not change during forced migration have significantly lower anxiety scores compared to those who report having it worsen.

#### 3.5.2 Social determinants

Model 2 demonstrated that social risk factors-related variables included in regression could statistically significantly predict anxiety levels and explained 16.9% of the variance in anxiety as evaluated by the BAI. Linear regression analysis showed that participants who reported improvements in relations with family members (*β* = −4.25) or relations that have remained unchanged (*β* = −5.32) have lower anxiety scores compared to those who report deterioration. Very similar is the situation with neighbors and coworkers’ relations. Females who reported improvements in relations (*β* = −5.84) or unchanged (*β* = −4.59) have lower anxiety scores compared to those who report deterioration. The same with relations with locals, individuals who report improvements in relations (*β* = −5.34) or unchanged (*β* = −4.70) have lower anxiety scores compared to those who report deterioration.

Moreover, respondents who declared experienced discrimination in the host country as refugees have significantly higher anxiety scores compared to those who did not. Additionally, it was found that females who have children under 18 years old (*β* = −2.06) have significantly lower anxiety scores compared to those who do not. Model 2 revealed that improved or stable relationships correlated considerably with reduced anxiety, while discrimination and not having children under 18 were linked to higher anxiety scores.

#### 3.5.3 Mental health determinants

Model 3 showed that variables related to mental health determinants included in regression could statistically significantly predict anxiety levels and explained 32.4% of the variance in anxiety as measured by the BAI. Linear regression analysis showed that there was an association between lowering self-reported emotional and psychological status (*β* = 4.49) and increasing anxiety scores. Also, there are associations between amotion-focused (*β* = 4.11), avoidant (*β* = 5.53) coping strategies and an increase in anxiety scores. Individuals with moderate (*β* = 6.67) or high (*β* = 10.50) perceived stress have significantly higher anxiety scores compared to those with low perceived stress. Model 2 revealed positive correlations between anxiety and ineffective amotion-focused and avoidant coping strategies, as well as negative correlations between anxiety and self-reported emotional and psychological status.

#### 3.5.4 Final model (including PHQ-9)

The final model defined those variables related to physical health, social, and mental health determinants included in the regression that could statistically significantly predict anxiety levels. It also explained 55.3% of the variance in anxiety as measured by the BAI. Depression was included as a covariate in the regression analysis because prior studies showed coexisting depression and anxiety. Linear regression analysis showed that there were associations between lowering self-reported emotional and psychological status (*β* = 2.44), lowering self-reported physical health (*β* = 1.43) and increasing anxiety scores. Moreover, respondents who reported their daily activities being hampered due to health issues (*β* = 3.25) have significantly higher anxiety scores compared to those who did not. Depression as a coexisting symptom of anxiety is the most significant factor in the model, indicating that an increase in the depression level is associated with an increase in anxiety score. The final model highlights the complex interactions between physical, social, and mental health in influencing anxiety. Depression, particularly, stands out as a critical determinant, reinforcing the need to consider both conditions together when assessing and treating anxiety.

## 4 Discussion

Since fleeing the motherland due to Russia’s large-scale invasion, Ukrainian female refugees have encountered numerous stressors, which have led to a notable increase in mental health issues, especially elevated anxiety levels. Anxiety disorders are some of the most common psychological conditions among refugee populations, often worsened by trauma, uncertainty, and the difficulties of resettling in a new country. This study’s aims were: (1) to evaluate the psychometric validity and reliability of the Ukrainian version of the BAI among Ukrainian female refugees in the Czech Republic (*N* = 919); (2) to determine the prevalence of anxiety; and (3) to identify key determinants for anxiety in this population.

### 4.1 The Ukrainian version of BAI

This study’s outcomes confirm the validity and reliability of the Ukrainian language versions of the BAI. Firstly, the results show that the BAI is a highly reliable tool for measuring anxiety severity. The BAI shows strong reliability across all models, as assessed by Cronbach’s *α* and McDonald’s *ω*. The one-factor model had the highest reliability. Becket al.’s two-factor model, the somatic and cognitive subscales had α = 0.89/ω = 0.89 and α = 0.84/ω = 0.85, respectively. Hewitt and Norton’s two-factor model increased the somatic subscale’s α to 0.90/ ω = 0.85, with the cognitive subscale at α = 0.85/ω = 0.85. Wetherell and Areán’s four-factor model offered more specificity, with the cognitive subscale at α = 0.85/ω = 0.85, autonomic at α = 0.73/ω = 0.74, neuromotor at α = 0.83/ω = 0.84, and panic at α = 0.77/ω = 0.79. All models exceeded accepted reliability thresholds, confirming the BAI as a reliable tool for assessing anxiety.

Secondly, the study found that 31.3% of female refugees experienced moderate symptoms of anxiety, while 22.1% experienced severe symptoms, raising concerns about their mental health. Such levels of anxiety are consistent with findings from other studies on refugee populations (Henkelmann et al., 2020; Nguyen et al., 2022; Zabarra et al., 2024; Essayagh et al., 2023).

Thirdly, the CFA conducted in this study indicated that a four-factor model, including cognitive, autonomic, neuromotor, and panic factors, was the most appropriate. Originally proposed by Wetherell and Areán (1997), the four-factor structure of the BAI has been consistently replicated across diverse populations and clinical settings. For example, Osman et al. (1997) reported an excellent fit for this model in a study involving undergraduates. Other examples include, the application of the four-factor model in cardiovascular and coronary artery disease patients (Clark et al., 2016; Rezaee et al., 2024).

### 4.2 Key anxiety determinants

The majority of Ukrainian female refugees in Czechia were an average age of 38. The majority were married or living with a partner, and more than two-thirds reported having children under 18. Most participants had a university education, lived in Ukrainian cities or towns, and were employed in Ukraine. Female refugees primarily came from the central, southern, and eastern regions of Ukraine. On average, they had been in the Czech Republic for 15 weeks before participating in the survey.

The study found that over half of the participants experienced moderate to concerning anxiety. Severe symptoms were common among Ukrainian female refugees, with 37.6% selecting ‘unable to relax’ and 42.3% selecting ‘fearing the worst’. Notable severe responses included ‘feeling nervous’ and ‘terrified’, as well as physical symptoms such as ‘heart racing’ and ‘feeling unsteady’ were also prevalent, affecting many at moderate to severe levels. The results align with findings from other studies on refugees, which also reported high levels of anxiety, particularly among women. Consistently, refugee populations showed a significant prevalence of severe anxiety symptoms, including physical manifestations such as heart racing, and emotional distress including fear and nervousness. A study among Syrian refugees in Ireland showed that 27.5% suffered from anxiety, for example, ‘feeling blue’ and ‘feeling hopeless about the future’ (Collins et al., 2022). A systematic review and meta-analysis of migrants in the Middle East and North Africa region showed 43% anxiety (Zabarra et al., 2024). In Morocco, anxiety prevalence among migrants was 39.1% (Essayagh et al., 2023). Overall, this demonstrates that refugees face numerous stressors that contribute to elevated anxiety levels. Anxiety disorders are therefore prevalent mental health conditions influenced by a complex interplay of physical and mental health determinants as well as social risk factors.

In more detail, the findings revealed that Ukrainian females whose physical health remained unchanged during the last month reported lower anxiety. Further, health deterioration due to war and forced migration significantly increased anxiety levels. Additionally, those requiring continuous medical supervision had higher anxiety scores. Also, daily activities hampered by health issues were linked to higher anxiety. According to another study with Syrian refugees in Ireland, participants who reported having chronic pain, were also more likely to have high anxiety levels (Collins et al., 2022). Therefore, decreasing self-reported physical health showed a strong correlation with increasing anxiety.

Regarding the social determinants, the analysis showed that participants who reported either improvements or stability in their relationships with family members had lower anxiety level compared to those who experienced issues. This pattern also extended to relationships with neighbors, coworkers, and locals. So, social support and networking are clearly important for the mental health of Ukrainian female refugees. Similarly, another study showed that participants in the lowest social support quintile had higher anxiety (Almeida et al., 2012). Additionally, in this study respondents who experienced discrimination in the host country, reported significantly higher anxiety. According to previous studies, discrimination is a strong predictor of increased anxiety among refugees. For example, increased symptoms of anxiety were predicted among females and individuals who experienced higher levels of perceived discrimination, as noted by Collins et al. (2022). Interestingly, according to this study, females with children under 18 years old showed significantly lower anxiety compared to those without children. This may be because having children gives Ukrainian females a clear purpose for their migration, such as ensuring their children’s safety and education. Additionally, they often assume the role of re-establishing a home in the host country, protecting family values, and preserving their national culture, which may offer them a greater sense of forced migration which can reduce anxiety.

In terms of mental health determinants, in this study, lower self-reported phyco-emotional status, as well as applying emotion-focused and avoidant coping strategies, were associated with increased anxiety. Few other studies have shown that emotion-focused and avoidance coping strategies were associated with psychological symptoms and self-reported poorer physical health in refugees. Ineffective coping strategies have been demonstrated to predispose refugees to mental health disorders, such as post-traumatic stress disorder, anxiety, and depression. Females who reported using self-blame, as a coping mechanism, had significantly higher levels of anxiety, according to Kelly et al. (2008). Another study showed that the use of these ineffective strategies tends to increase social anxiety, further impairing the individual’s ability to function effectively in social contexts (Thomasson and Psouni, 2010). Additionally, this study revealed that moderate and high perceived stress led to higher anxiety scores, which are similar to a study among migrants in Morocco (Essayagh et al., 2023).

Regarding the socio-demographic factors, this study with Ukrainian female refugees showed that each increase in age group correlated with a decrease in anxiety levels, the same as the total BAI score was found to correlate negatively with age in Wetherell and Areán study (1997). Additionally, the finding of this study revealed that females with unchanged economic status during forced migration reported lower anxiety. This was echoed in another study among Ukrainians who were either refugees in other countries or residents of Ukraine’s territory, it was reported that financial status had a statistically significant impact on anxiety (Kurapov et al., 2023). Correlations were found between higher levels of anxiety and low income (Essayagh et al., 2023) as well as financial strain (Almeida et al., 2012) in other studies.

This study overall has identified some determinants contributing to anxiety among refugees. The final model in the analysis of this study, identified four key factors contributing to heightened anxiety: poor self-reported mental health; poor self-reported physical health; limitations in daily activities due to health issues; and, most notably, depression as a critical comorbid condition. These findings highlight the importance of comprehensive approaches that aim to address both anxiety and depression together. Additionally, this study is the first to empirically and theoretically validate a four-factor structure model for the Ukrainian version of the BAI, and the first to identify the relationship between self-reported social, physical, and mental health factors and anxiety among Ukrainian female refugees in the Czech Republic. However, several limitations should be noted. The use of online questionnaires limited participation to more educated females with strong digital skills. Future research should aim to include older females with lower digital literacy. Additionally, self-reports may be subject to social desirability bias, however, in this study strategies such as anonymity, self-administered questionnaires, and forced-choice items were employed to mitigate this issue.

## 5 Conclusion

This study evaluated the psychometric validity and reliability of the Ukrainian version of the BAI while also identifying key determinants of anxiety among Ukrainian female refugees in the Czech Republic. The findings confirmed that the BAI is a reliable tool with strong reliability across various models. The study revealed that over half of the participants experienced moderate to severe anxiety, with poor physical and mental health, limitations in daily activities, and depression being significant contributors to elevated anxiety levels. Social support and economic stability were associated with lower anxiety, while discrimination and applying ineffective coping strategies, such as avoidance, were linked to increased anxiety.

The study also highlighted that females with children under 18 reported lower anxiety, likely due to the sense of purpose provided by caring for their children. Notably, socio-demographic factors, such as age and stable economic status, correlated with reduced anxiety. The study is the first to empirically validate the four-factor structure of the Ukrainian version of BAI and its association with social, physical, and mental health determinants in female refugee populations. These findings highlight the need for comprehensive interventions that address the diverse determinants to effectively reduce the prevalence of comorbid anxiety and depression.

## Data Availability

The data will be available upon reasonable request. Third parties may not be provided access to data if doing so would violate applicable EU data protection laws as well as national data protection laws in the project’s member nations. The process can be started by interested researchers contacting corresponding author.
