# Supplementary material for: Psychometric assessment of the Beck anxiety inventory and key anxiety determinants among Ukrainian female refugees in the Czech Republic
Source: Front Psychol. 2025 Jan 15;15:1529718. doi: 10.3389/fpsyg.2024.1529718 (PMC11774926; doi:10.3389/fpsyg.2024.1529718)
Supplement: Supplementary file 1 [file Data_Sheet_1.docx]

**Appendix 1.** The Ukrainian version of the BAI

**ШКАЛА ТРИВОГИ БЕКА (BAI)**

Нижче наведений перелік загальних симптомів тривоги. Будь ласка, уважно прочитайте кожний пункт. Зазначте, як сильно цей симптом непокоїв Вас за останній місяць, включаючи сьогодні, шляхом зазначення у відповідному місці у стовпчику, зазначеному після кожного симптому.

|  | **Зовсім не турбувало** | **Злегка турбувало** | **Середньо турбувало** | **Сильно турбувало** |
| --- | --- | --- | --- | --- |
| 1. **Відчуття оніміння або поколювання в тілі** |  |  |  |  |
| 1. **Відчуття жару** |  |  |  |  |
| 1. **Тремтіння в ногах** |  |  |  |  |
| 1. **Неможливість розслабитися** |  |  |  |  |
| 1. **Страх, що станеться найгірше** |  |  |  |  |
| 1. **Головокружіння або відчуття легкості в голові** |  |  |  |  |
| 1. **Прискорене серцебиття** |  |  |  |  |
| 1. **Нестійкість, нестабільність** |  |  |  |  |
| 1. **Відчуття жаху або страху** |  |  |  |  |
| 1. **Дратівливість** |  |  |  |  |
| 1. **Відчуття задухи** |  |  |  |  |
| 1. **Тремтіння в руках** |  |  |  |  |
| 1. **Хиткість/нерівність ходи** |  |  |  |  |
| 1. **Страх втрати контролю** |  |  |  |  |
| 1. **Труднощі з диханням** |  |  |  |  |
| 1. **Страх смерті** |  |  |  |  |
| 1. **Переляк** |  |  |  |  |
| 1. **Шлунково-кишкові розлади** |  |  |  |  |
| 1. **Непритомність/Відчуття легкості в голові** |  |  |  |  |
| 1. **Припливи крові до обличчя** |  |  |  |  |
| 1. **Посилене потовиділення або виділення холодного поту** |  |  |  |  |

**Appendix 2.** Socio-demographic, self-reported general health, emotions, and psychological status characteristics of Ukrainian female refugees in the Czech Republic (n = 919)

|  | **Frequency** | **%** |
| --- | --- | --- |
| **Socio-demographic characteristics:** |  |  |
| Marriage status |  |  |
| Single | 141 | 15.8 |
| Married | 610 | 68.4 |
| Divorced/separated | 141 | 15.8 |
| Have children under 18 years old |  |  |
| No | 268 | 29.3 |
| Yes | 646 | 70.7 |
| Age Groups |  |  |
| Under 30 | 198 | 21.9 |
| 30-40 | 379 | 41.9 |
| 40+ | 328 | 36.2 |
| Economic Financial Status Now |  |  |
| become better | 80 | 8.8 |
| stayed the same | 141 | 15.4 |
| become worst | 692 | 75.8 |
| Economic Financial Status Before War |  |  |
| Not enough money for food | 10 | 1.1 |
| Enough money for food, buying clothes was difficult | 176 | 19.3 |
| Enough money for food, clothes, making savings | 618 | 67.8 |
| May buying expensive things, making big savings | 108 | 11.8 |
| Highest Level of Education |  |  |
| Secondary/Vocational | 76 | 8.3 |
| Undergraduate/Bachelor | 150 | 16.4 |
| Graduate/PhD | 687 | 75.2 |
| Employed in the Czech Republic |  |  |
| No | 637 | 69.3 |
| Yes | 277 | 30.1 |
| **Physical health factors:** |  |  |
| Necessary Medical Aid |  |  |
| Enough | 319 | 42.3 |
| Not enough | 379 | 50.2 |
| Difficult to say/n/a | 57 | 7.5 |
| Changes in Physical Health During the Last Month |  |  |
| got better | 84 | 9.2 |
| stayed the same | 575 | 62.9 |
| got worst | 255 | 27.9 |
| Self-Reported Physical Health (SRPH) |  |  |
| Very Good | 35 | 3.8 |
| Good | 358 | 39.2 |
| Fair | 427 | 46.8 |
| Bad | 74 | 8.1 |
| Very Bad | 19 | 2.1 |
| Lost Health due to the war |  |  |
| Yes | 41 | 4.5 |
| No | 744 | 81.0 |
| Difficult to say | 127 | 13.9 |
| Necessary of continuously medical supervision |  |  |
| Yes | 161 | 17.7 |
| No | 593 | 65.1 |
| Difficult to say | 157 | 17.2 |
| Registered with GP |  |  |
| Yes | 252 | 27.7 |
| No, still looking for | 337 | 37.0 |
| No, not looking, lost hope to find | 196 | 21.5 |
| No, not looking, using UA medical service | 126 | 13.8 |
| Limited in daily activities due to ill health |  |  |
| No | 639 | 70.0 |
| Yes, to some extend | 234 | 25.6 |
| Yes, much | 21 | 2.3 |
| Disability | 19 | 2.1 |
| Chronic diseases |  |  |
| No | 378 | 41.6 |
| Yes, one | 318 | 35.0 |
| Yes, two | 142 | 15.6 |
| Yes, three or more | 70 | 7.7 |
| **Social health determinants** |  |  |
| Self-Reported Emotional and Psychological Status (SREPS) |  |  |
| Very Good | 11 | 1.2 |
| Good | 113 | 12.4 |
| Fair | 480 | 52.7 |
| Bad | 237 | 26.0 |
| Very Bad | 70 | 7.7 |
| Have someone to share personal problem with |  |  |
| Yes | 592 | 65.1 |
| No | 124 | 13.6 |
| Difficult to say | 193 | 21.2 |
| Relationships with family members |  |  |
| got better | 200 | 22.1 |
| stayed the same | 559 | 61.8 |
| got worst | 145 | 16.0 |
| Relationships with friends |  |  |
| got better | 94 | 10.5 |
| stayed the same | 624 | 69.7 |
| got worst | 177 | 19.8 |
| Relationships with colleagues/neighbors |  |  |
| got better | 72 | 8.2 |
| stayed the same | 658 | 74.6 |
| got worst | 152 | 17.2 |
| Relationships with locals |  |  |
| got better | 164 | 18.5 |
| stayed the same | 673 | 76.0 |
| got worst | 48 | 5.4 |
| Attending meetings organized for refugees |  |  |
| No | 342 | 37.7 |
| No, but plan to | 154 | 17.0 |
| Yes, but rarely | 305 | 33.6 |
| Yes, often | 106 | 11.7 |
| Participating in Ukrainian religious communities |  |  |
| Yes | 86 | 9.8 |
| No, but plan to | 80 | 9.1 |
| Yes | 711 | 81.1 |
| Cultural differences | |  |
| Yes | 106 | 11.5 |
| No | 813 | 88.5 |
| Discrimination | |  |
| Yes | 96 | 10.4 |
| No | 823 | 89.6 |
| Necessary Phycological Aid |  |  |
| Enough | 319 | 42.3 |
| Not enough | 379 | 50.2 |
| Difficult to say/n/a | 57 | 7.5 |
| **Coping strategies (BRIEF-COPE)** |  |  |
| Problem Focused Strategy |  |  |
| Lower then 2.5 | 284 | 35.5 |
| Higher then 2.5 | 517 | 64.5 |
| Emotion Focused Strategy |  |  |
| Lower then 2.5 | 556 | 70.5 |
| Higher then 2.5 | 233 | 29.5 |
| Avoidant Strategy |  |  |
| Lower then 2.5 | 634 | 78.4 |
| Higher then 2.5 | 175 | 21.6 |
| **Perceived stress (PSS-14)** |  |  |
| Low (0-18) | 34 | 4.4 |
| Moderate (19-37) | 577 | 75.2 |
| High (38-56) | 156 | 20.3 |
| **Depression (PHQ-9)** |  |  |
| Minimal | 134 | 16.6 |
| Mild | 241 | 29.9 |
| Moderate | 205 | 25.5 |
| Moderately Severe | 141 | 17.5 |
| Severe | 84 | 10.4 |
| **Anxiety (BAI)** |  |  |
| Minimal | 71 | 9.2 |
| Mild | 161 | 20.9 |
| Moderate | 213 | 27.7 |
| Severe | 325 | 42.2 |

**Appendix 3.** The reliability of the BAI (measured with Cronbach’s α and McDonald’s ω) thought factor models.

| **Factor Model** | **Items** | **Cronbach’s α** | **McDonald’s ω** |
| --- | --- | --- | --- |
| One-factor | 1-21 | 0.924 | 0.925 |
| Two-factor  (Beck et al.'s model) | Somatic (1, 2, 3, 6, 7, 8, 12, 13, 17, 19, 20, 21) | 0.885 | 0.887 |
|  | Cognitive (4, 5, 9, 10, 11, 14, 15, 16, 18) | 0.843 | 0.845 |
| Two-factor  (Hewitt & Norton's model) | Somatic (1, 2, 3, 6, 7, 8, 11, 12, 13, 15, 18, 19, 20, 21) | 0.903 | 0.905 |
|  | Cognitive (4, 5, 9, 10, 14, 16, 17) | 0.849 | 0.851 |
| Four-factor  (Wetherell & Areán model) | Cognitive (4, 5, 9, 10, 14, 16, 17) | 0.849 | 0.851 |
|  | Autonomic (2, 18, 20, 21) | 0.729 | 0.737 |
|  | Neuromotor (1, 3, 6, 8, 12, 13, 19) | 0.832 | 0.835 |
|  | Panic (7, 11, 15) | 0.769 | 0.785 |

**Appendix 4.** Confirmatory factor analysis measurement models

Measurement Model 1 data:

| **Latent Variable** | **Observed Variable** | **Estimate** | **SE** | **95% CI** | **β** | **z** | **p-value** |
| --- | --- | --- | --- | --- | --- | --- | --- |
| **All Factors** | Numbness or tingling | 1.000 | 0.0000 | 1.000 - 1.000 | 0.643 | - | - |
|  | Feeling hot | 1.001 | 0.0251 | 0.952 - 1.050 | 0.644 | 39.9 | < .001 |
|  | Wobbliness in legs | 1.125 | 0.0268 | 1.073 - 1.178 | 0.724 | 42.0 | < .001 |
|  | Unable to relax | 1.081 | 0.0261 | 1.029 - 1.132 | 0.695 | 41.4 | < .001 |
|  | Fear of worst happening | 1.131 | 0.0263 | 1.080 - 1.183 | 0.727 | 43.1 | < .001 |
|  | Dizzy or lightheaded | 1.054 | 0.0252 | 1.005 - 1.104 | 0.678 | 41.9 | < .001 |
|  | Heart pounding/racing | 1.133 | 0.0257 | 1.082 - 1.183 | 0.728 | 44.0 | < .001 |
|  | Unsteady | 1.091 | 0.0256 | 1.041 - 1.141 | 0.701 | 42.6 | < .001 |
|  | Terrified or afraid | 1.170 | 0.0262 | 1.119 - 1.221 | 0.752 | 44.6 | < .001 |
|  | Nervous | 0.856 | 0.0238 | 0.809 - 0.903 | 0.550 | 35.9 | < .001 |
|  | Feeling of choking | 1.211 | 0.0272 | 1.157 - 1.264 | 0.778 | 44.5 | < .001 |
|  | Hands trembling | 1.142 | 0.0264 | 1.090 - 1.194 | 0.734 | 43.3 | < .001 |
|  | Shaky / unsteady | 1.124 | 0.0270 | 1.071 - 1.177 | 0.723 | 41.6 | < .001 |
|  | Fear of losing control | 1.024 | 0.0250 | 0.975 - 1.073 | 0.658 | 41.0 | < .001 |
|  | Difficulty in breathing | 1.244 | 0.0279 | 1.190 - 1.299 | 0.800 | 44.7 | < .001 |
|  | Fear of dying | 0.994 | 0.0255 | 0.944 - 1.044 | 0.639 | 39.0 | < .001 |
|  | Scared | 1.121 | 0.0261 | 1.070 - 1.173 | 0.721 | 43.0 | < .001 |
|  | Indigestion | 0.831 | 0.0240 | 0.784 - 0.879 | 0.535 | 34.6 | < .001 |
|  | Faint / lightheaded | 1.009 | 0.0267 | 0.957 - 1.061 | 0.649 | 37.7 | < .001 |
|  | Face flushed | 0.950 | 0.0250 | 0.901 - 0.999 | 0.611 | 38.0 | < .001 |
|  | Hot/cold sweats | 1.031 | 0.0253 | 0.982 - 1.081 | 0.663 | 40.8 | < .001 |

Measurement Model 2 data:

| **Latent** | **Observed** | **Estimate** | **SE** | **95% CI** | **β** | **z** | **p** |
| --- | --- | --- | --- | --- | --- | --- | --- |
| **Cognitive** | Unable to relax | 1.000 | 0.0000 | 1.000-1.000 | 0.714 |  |  |
|  | Fear of worst happening | 1.046 | 0.0239 | 0.999-1.093 | 0.747 | 43.8 | < .001 |
|  | Terrified or afraid | 1.084 | 0.0233 | 1.038-1.130 | 0.774 | 46.5 | < .001 |
|  | Nervous | 0.791 | 0.0217 | 0.749-0.834 | 0.565 | 36.5 | < .001 |
|  | Feeling of choking | 1.120 | 0.0239 | 1.074-1.167 | 0.800 | 47.0 | < .001 |
|  | Fear of losing control | 0.949 | 0.0225 | 0.905-0.993 | 0.678 | 42.2 | < .001 |
|  | Difficulty in breathing | 1.152 | 0.0245 | 1.104-1.200 | 0.823 | 47.1 | < .001 |
|  | Fear of dying | 0.923 | 0.0228 | 0.878-0.967 | 0.659 | 40.4 | < .001 |
|  | Indigestion | 0.768 | 0.0217 | 0.726-0.811 | 0.549 | 35.4 | < .001 |
| **Somatic** | Numbness or tingling | 1.000 | 0.0000 | 1.000-1.000 | 0.654 |  |  |
|  | Feeling hot | 1.001 | 0.0251 | 0.952-1.050 | 0.654 | 39.9 | < .001 |
|  | Wobbliness in legs | 1.124 | 0.0267 | 1.071-1.176 | 0.735 | 42.0 | < .001 |
|  | Dizzy or lightheaded | 1.054 | 0.0251 | 1.005-1.103 | 0.689 | 42.0 | < .001 |
|  | Heart pounding/racing | 1.133 | 0.0257 | 1.083-1.184 | 0.741 | 44.0 | < .001 |
|  | Unsteady | 1.092 | 0.0256 | 1.041-1.142 | 0.714 | 42.6 | < .001 |
|  | Hands trembling | 1.142 | 0.0264 | 1.090-1.194 | 0.747 | 43.3 | < .001 |
|  | Shaky/unsteady | 1.124 | 0.0270 | 1.071-1.177 | 0.735 | 41.6 | < .001 |
|  | Scared | 1.122 | 0.0262 | 1.071-1.174 | 0.734 | 42.8 | < .001 |
|  | Faint/lightheaded | 1.009 | 0.0267 | 0.956-1.061 | 0.660 | 37.8 | < .001 |
|  | Face flushed | 0.950 | 0.0249 | 0.901-0.999 | 0.621 | 38.1 | < .001 |
|  | Hot/cold sweats | 1.031 | 0.0252 | 0.982-1.081 | 0.674 | 40.9 | < .001 |

Variances and Covariances data:

| **Variable 1** | **Variable 2** | **Estimate** | **SE** | **95% CI** | **β** | **z** | **p-value** |
| --- | --- | --- | --- | --- | --- | --- | --- |
| **Cognitive** | **Somatic** | 0.426 | 0.0104 | 0.406 - 0.447 | 0.912 | 41.0 | < .001 |

Measurement Model 3 data:

| **Latent** | **Observed** | **Estimate** | **SE** | **Lower** | **Upper** | **β** | **z** | **p** |
| --- | --- | --- | --- | --- | --- | --- | --- | --- |
| **Cognitive** | V25.4R | 1.000 | 0.0000 | 1.000 | 1.000 | 0.763 |  |  |
|  | V25.5R | 1.040 | 0.0240 | 0.993 | 1.087 | 0.794 | 43.3 | < .001 |
|  | V25.9R | 1.081 | 0.0235 | 1.035 | 1.127 | 0.825 | 46.1 | < .001 |
|  | V25.10R | 0.793 | 0.0219 | 0.750 | 0.835 | 0.605 | 36.1 | < .001 |
|  | V25.14R | 0.959 | 0.0230 | 0.913 | 1.004 | 0.732 | 41.6 | < .001 |
|  | V25.16R | 0.929 | 0.0228 | 0.885 | 0.974 | 0.709 | 40.7 | < .001 |
|  | V25.17R | 1.045 | 0.0234 | 1.000 | 1.091 | 0.798 | 44.7 | < .001 |
| **Somatic** | V25.1R | 1.000 | 0.0000 | 1.000 | 1.000 | 0.663 |  |  |
|  | V25.2R | 0.999 | 0.0249 | 0.951 | 1.048 | 0.663 | 40.1 | < .001 |
|  | V25.3R | 1.120 | 0.0266 | 1.068 | 1.172 | 0.743 | 42.1 | < .001 |
|  | V25.6R | 1.053 | 0.0250 | 1.004 | 1.102 | 0.698 | 42.1 | < .001 |
|  | V25.7R | 1.134 | 0.0257 | 1.084 | 1.185 | 0.752 | 44.1 | < .001 |
|  | V25.8R | 1.095 | 0.0258 | 1.044 | 1.145 | 0.726 | 42.5 | < .001 |
|  | V25.11R | 1.201 | 0.0269 | 1.148 | 1.253 | 0.797 | 44.7 | < .001 |
|  | V25.12R | 1.137 | 0.0262 | 1.086 | 1.189 | 0.755 | 43.5 | < .001 |
|  | V25.13R | 1.120 | 0.0268 | 1.068 | 1.173 | 0.743 | 41.8 | < .001 |
|  | V25.15R | 1.234 | 0.0275 | 1.180 | 1.288 | 0.818 | 44.8 | < .001 |
|  | V25.18R | 0.833 | 0.0240 | 0.786 | 0.880 | 0.553 | 34.7 | < .001 |
|  | V25.19R | 1.008 | 0.0265 | 0.956 | 1.060 | 0.669 | 38.0 | < .001 |
|  | V25.20R | 0.950 | 0.0248 | 0.902 | 0.999 | 0.630 | 38.3 | < .001 |
|  | V25.21R | 1.031 | 0.0251 | 0.982 | 1.081 | 0.684 | 41.1 | < .001 |

Variances and Covariances data:

| **Variable 1** | **Variable 2** | **Estimate** | **SE** | **95% CI** | **β** | **z** | **p-value** |
| --- | --- | --- | --- | --- | --- | --- | --- |
| **Cognitive** | **Somatic** | 0.383 | 0.00949 | 0.364 - 0.401 | 0.756 | 40.3 | < .001 |

Measurement Model 4 data:

| **Latent Variable** | **Observed Variable** | **Estimate** | **SE** | **95% CI** | **β** | **z** | **p-value** |
| --- | --- | --- | --- | --- | --- | --- | --- |
| **Cognitive** | Unable to relax | 1.000 | 0.0000 | 1.000 - 1.000 | 0.763 | - | - |
|  | Fear of worst happening | 1.040 | 0.0240 | 0.993 - 1.087 | 0.794 | 43.3 | < .001 |
|  | Terrified or afraid | 1.081 | 0.0234 | 1.035 - 1.127 | 0.825 | 46.1 | < .001 |
|  | Nervous | 0.792 | 0.0219 | 0.749 - 0.835 | 0.605 | 36.1 | < .001 |
|  | Fear of losing control | 0.959 | 0.0230 | 0.914 - 1.004 | 0.732 | 41.6 | < .001 |
|  | Fear of dying | 0.930 | 0.0228 | 0.885 - 0.975 | 0.710 | 40.7 | < .001 |
|  | Scared | 1.045 | 0.0234 | 0.999 - 1.091 | 0.798 | 44.7 | < .001 |
| **Autonomic** | Feeling hot | 1.000 | 0.0000 | 1.000 - 1.000 | 0.743 | - | - |
|  | Indigestion | 0.828 | 0.0239 | 0.782 - 0.875 | 0.616 | 34.6 | < .001 |
|  | Face flushing | 0.946 | 0.0249 | 0.897 - 0.994 | 0.703 | 38.0 | < .001 |
|  | Hot/cold sweats | 1.030 | 0.0256 | 0.980 - 1.080 | 0.766 | 40.3 | < .001 |
| **Neuromotor** | Numbness or tingling | 1.000 | 0.0000 | 1.000 - 1.000 | 0.673 | - | - |
|  | Wobbliness in legs | 1.121 | 0.0267 | 1.069 - 1.173 | 0.754 | 42.0 | < .001 |
|  | Dizzy or lightheaded | 1.055 | 0.0251 | 1.006 - 1.104 | 0.710 | 42.0 | < .001 |
|  | Unsteady | 1.097 | 0.0259 | 1.046 - 1.147 | 0.738 | 42.3 | < .001 |
|  | Shaky/unsteady | 1.121 | 0.0269 | 1.068 - 1.174 | 0.754 | 41.7 | < .001 |
|  | Hands trembling | 1.139 | 0.0263 | 1.088 - 1.190 | 0.766 | 43.4 | < .001 |
|  | Faint/lightheaded | 1.008 | 0.0266 | 0.956 - 1.061 | 0.678 | 37.9 | < .001 |
| **Panic** | Heart pounding/racing | 1.000 | 0.0000 | 1.000 - 1.000 | 0.793 | - | - |
|  | Feeling of choking | 1.048 | 0.0211 | 1.006 - 1.089 | 0.831 | 49.7 | < .001 |
|  | Difficulty breathing | 1.077 | 0.0217 | 1.034 - 1.119 | 0.854 | 49.7 | < .001 |

Variances and Covariances:

| **Variable 1** | **Variable 2** | **Estimate** | **SE** | **95% CI** | **β** | **z** | **p-value** |
| --- | --- | --- | --- | --- | --- | --- | --- |
| **Cognitive** | **Autonomic** | 0.360 | 0.0108 | 0.339 - 0.381 | 0.634 | 33.2 | < .001 |
| **Cognitive** | **Neuromotor** | 0.390 | 0.0101 | 0.370 - 0.410 | 0.759 | 38.4 | < .001 |
| **Cognitive** | **Panic** | 0.442 | 0.0110 | 0.420 - 0.463 | 0.730 | 40.1 | < .001 |
| **Autonomic** | **Neuromotor** | 0.423 | 0.0112 | 0.401 - 0.445 | 0.846 | 37.6 | < .001 |
| **Autonomic** | **Panic** | 0.474 | 0.0131 | 0.448 - 0.499 | 0.804 | 36.2 | < .001 |
| **Neuromotor** | **Panic** | 0.484 | 0.0116 | 0.461 - 0.506 | 0.907 | 41.8 | < .001 |

**
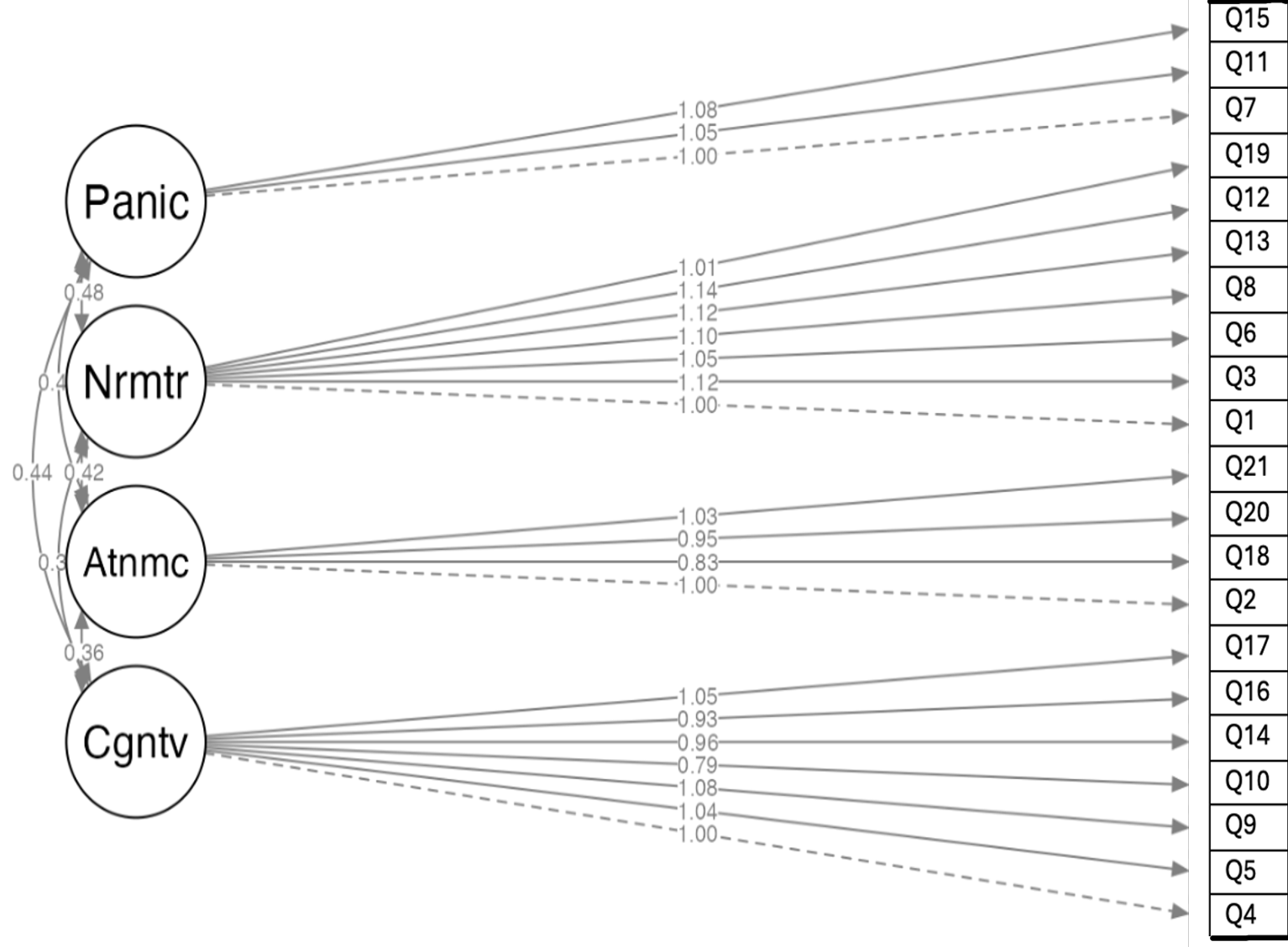
**

Measurement Model 5 data:

| **Latent Variable** | **Observed Variable** | **Estimate** | **SE** | **95% CI** | **β** | **z** | **p-value** |
| --- | --- | --- | --- | --- | --- | --- | --- |
| **Cognitive** | Unable to relax | 1.000 | 0.0000 | 1.000 - 1.000 | 0.763 | - | - |
|  | Fear of worst happening | 1.040 | 0.0240 | 0.993 - 1.087 | 0.794 | 43.3 | < .001 |
|  | Terrified or afraid | 1.081 | 0.0234 | 1.035 - 1.127 | 0.825 | 46.1 | < .001 |
|  | Nervous | 0.792 | 0.0219 | 0.749 - 0.835 | 0.605 | 36.1 | < .001 |
|  | Fear of losing control | 0.959 | 0.0230 | 0.914 - 1.004 | 0.732 | 41.6 | < .001 |
|  | Fear of dying | 0.930 | 0.0228 | 0.885 - 0.974 | 0.710 | 40.7 | < .001 |
|  | Scared | 1.045 | 0.0234 | 0.999 - 1.091 | 0.798 | 44.7 | < .001 |
| **Autonomic** | Feeling hot | 1.000 | 0.0000 | 1.000 - 1.000 | 0.743 | - | - |
|  | Indigestion | 0.828 | 0.0239 | 0.781 - 0.875 | 0.615 | 34.7 | < .001 |
|  | Face flushed | 0.945 | 0.0249 | 0.897 - 0.994 | 0.703 | 38.0 | < .001 |
|  | Hot/cold sweats | 1.030 | 0.0256 | 0.980 - 1.080 | 0.766 | 40.3 | < .001 |
| **Neuromotor** | Numbness or tingling | 1.000 | 0.0000 | 1.000 - 1.000 | 0.673 | - | - |
|  | Wobbliness in legs | 1.121 | 0.0267 | 1.069 - 1.173 | 0.754 | 42.0 | < .001 |
|  | Dizzy or lightheaded | 1.055 | 0.0251 | 1.005 - 1.104 | 0.710 | 42.0 | < .001 |
|  | Unsteady | 1.096 | 0.0259 | 1.046 - 1.147 | 0.738 | 42.4 | < .001 |
|  | Shaky/unsteady | 1.121 | 0.0268 | 1.068 - 1.173 | 0.754 | 41.8 | < .001 |
|  | Hands trembling | 1.139 | 0.0262 | 1.087 - 1.190 | 0.766 | 43.4 | < .001 |
|  | Faint/lightheaded | 1.008 | 0.0266 | 0.956 - 1.060 | 0.678 | 37.9 | < .001 |
| **Panic** | Heart pounding/racing | 1.000 | 0.0000 | 1.000 - 1.000 | 0.793 | - | - |
|  | Feeling of choking | 1.047 | 0.0211 | 1.006 - 1.089 | 0.831 | 49.7 | < .001 |
|  | Difficulty in breathing | 1.076 | 0.0217 | 1.034 - 1.119 | 0.854 | 49.7 | < .001 |
| **General** | **Cognitive** | 1.000 | 0.0000 | 1.000 - 1.000 | 0.770 | - | - |
|  | **Autonomic** | 1.076 | 0.0284 | 1.021 - 1.132 | 0.851 | 37.9 | < .001 |
|  | **Neuromotor** | 1.124 | 0.0312 | 1.063 - 1.185 | 0.982 | 36.0 | < .001 |
|  | **Panic** | 1.261 | 0.0307 | 1.201 - 1.321 | 0.934 | 41.1 | < .001 |

Variances and Covariances data:

| **Variable 1** | **Variable 2** | **Estimate** | **SE** | **95% CI** | **β** | **z** | **p-value** |
| --- | --- | --- | --- | --- | --- | --- | --- |
| **Cognitive** | **Cognitive** | 0.2371 | 0.0122 | 0.21310 - 0.2610 | 0.4069 | 19.40 | < .001 |
| **Autonomic** | **Autonomic** | 0.1524 | 0.0174 | 0.11834 - 0.1864 | 0.2758 | 8.77 | < .001 |
| **Neuromotor** | **Neuromotor** | 0.0163 | 0.0102 | -0.00380 - 0.0363 | 0.0359 | 1.59 | 0.112 |
| **Panic** | **Panic** | 0.0797 | 0.0177 | 0.04502 - 0.1144 | 0.1268 | 4.50 | < .001 |
| **General** | **General** | 0.3455 | 0.0127 | 0.32056 - 0.3704 | 1.0000 | 27.17 | < .001 |

**Appendix 5.** The correlation Matrix (Pearson's *r*) of the BRIEF-COPE (three-factor structure: problem-focused, emotional-focused and avoidant strategies), Self-Reported Physical Health (SRPH) and Self-Reported Emotional and Psychological Status (SREPS), Perceived Stress Scale (PSS-14), BAI (Beck Anxiety Inventory) and PHQ-9 (Patient Health Questionnaire-9).

|  | 1 | 2 | 3 | 4 | 5 | 6 | 7 | 8 |
| --- | --- | --- | --- | --- | --- | --- | --- | --- |
| **1. Problem Focused (BRIEF-COPE)** | — |  |  |  |  |  |  |  |
| **2. Emotion Focused (BRIEF-COPE)** | 0.615*** | — |  |  |  |  |  |  |
| **3. Avoidant**  **(BRIEF-COPE)** | 0.167*** | 0.343*** | — |  |  |  |  |  |
| **4. SPH** | -0.055 | 0.018 | 0.182*** | — |  |  |  |  |
| **5. SREPS** | -0.027 | 0.103** | 0.403*** | 0.324*** | — |  |  |  |
| **6. Total PSS-14** | -0.131 *** | 0.070 | 0.479 *** | 0.288 *** | 0.612 *** | — |  |  |
| **7. Total BAI** | 0.018 | 0.233 *** | 0.473 *** | 0.394 *** | 0.494 *** | 0.525 *** | — |  |
| **8. Total PHQ-9** | -0.024 | 0.216*** | 0.531 *** | 0.347 *** | 0.565 *** | 0.595 *** | 0.687 *** | — |

* p < .05, ** p < .01, *** p < .001

**Appendix 6.** Physical health determinants, barriers to adaptation, social health determinants, coping strategies and perceived stress associations

* p < .05, ** p < .01, *** p < .001

|  | **Model 1**  **(physical health determinants)**  ***B* (95%CI)** | **Model 3**  **(social determinants)**  ***B* (95%CI)** | **Model 3**  **(mental health determinants)**  ***B* (95%CI)** | **Final Model**  **(including PHQ-9)**  ***B* (95%CI)** |
| --- | --- | --- | --- | --- |
| R^2^ | 0.275 | 0.169 | 0.324 | 0.553 |
| **Socio-demographic characteristics:** |  |  |  |  |
| **Age** | **-1.566 (-2.84; -0.29) *** | 0.1688 (-1.09; 1.43) | -0.6257 (-2.01; 0.76) | -0.1839 (-1.23; 0.86) |
| **Have children under 18 years old** |  |  |  |  |
| No | Ref | Ref | Ref | Ref |
| Yes | -0.260 (-2.30; 1.78) | **-2.0576 (-4.09; -0.03) *** | -0.8854 (-3.12; 1.35) | -0.1848 (-1.85; 1.48) |
| **Economic Financial Status Now** |  |  |  |  |
| become better | -2.073 (-5.57; 1.42) | **-4.3416 (-7.73; -0.95) *** | -1.9492 (-6.18; 2.28) | -1.6315 (-4.40; 1.14) |
| stayed the same | **-2.854 (-5.39; -0.32) *** | -1.9909 (-4.57; 0.59) | 1.6670 (-1.23; 4.57) | -0.4322 (-2.53; 1.67) |
| become worst | Ref | Ref | Ref | Ref |
| **Employed in the Czech Republic** |  |  |  |  |
| No | Ref | Ref | Ref | Ref |
| Yes | -1.780 (-3.78; 0.22) | -1.9825 (-3.99; 0.03) | -2.0617 (-4.35; 0.23) | -0.3448 (-2.01; 1.32) |
| **Physical health determinants:** |  |  |  |  |
| **Necessary Medical Aid** |  |  |  |  |
| Not enough | Ref |  |  |  |
| Enough | -1.912 (-3.97; 0.14) |  |  |  |
| Difficult to say/n/a | -2.049 (-5.64; 1.54) |  |  |  |
| **Changes in Physical Health During the Last Month** |  |  |  |  |
| got better | -2.564 (-6.03; 0.91) |  |  | 0.4871 (-2.46; 3.44) |
| stayed the same | **-2.789 (-4.95; -0.63) *** |  |  | 0.7797 (-1.11; 2.6) |
| got worst | Ref |  |  | Ref |
| **Self-Reported Physical Health (SRPH)** |  |  |  |  |
| Very Good | Ref |  |  | Ref |
| Good | 4.969 (-0.423; 10.36) |  |  | 1.3234 (-2.77; 5.42) |
| Fair | **8.487 (2.91; 14.07) **** |  |  | 2.6140 (-1.65; 6.87) |
| Bad | **13.731 (7.08; 20.38) ***** |  |  | 4.4395 (-0.95; 9.83) |
| Very Bad | **17.158 (8.29; 26.02) ***** |  |  | 5.9755 (-1.88; 13.83) |
| **Lost Health due to the war** |  |  |  |  |
| No | Ref |  |  | Ref |
| Yes | **6.806 (2.51; 11.11) **** |  |  | 3.4855 (-0.02; 6.99) |
| Difficult to say | 2.19 (-0.54; 4.91) |  |  | 1.1306 (-1.05; 3.31) |
| **Necessary of continuously medical supervision** |  |  |  |  |
| No | Ref |  |  | Ref |
| Yes | 2.661 (-0.14; 5.46) |  |  | 1.2955 (-0.95; 3.54) |
| Difficult to say | 1.672 (-0.93; 4.27) |  |  | 0.6673 (-1.46; 2.80) |
| **Registered with family doctor** |  |  |  |  |
| Yes | Ref |  |  |  |
| No, still looking for | 1.71 (-0.62; 4.04) |  |  |  |
| No, not looking, lost hope to find | 2.44 (-0.26; 5.14) |  |  |  |
| No, not looking, using UA medical service | 1.03 (-1.98; 4.04) |  |  |  |
| **Limited in daily activities due to ill health** |  |  |  |  |
| No | Ref |  |  | Ref |
| Yes | **3.757 (1.42; 6.09) **** |  |  | **3.2451 (1.27; 5.22) **** |
| **Chronic diseases** |  |  |  |  |
| No | Ref |  |  |  |
| Yes, 1 | 1.505 (-0.69; 3.70) |  |  |  |
| Yes, 2 | **-1.743 (-4.68; 1.20) *** |  |  |  |
| Yes, 3 or more | **0.803 (-3.37; 4.98) *** |  |  |  |
| **COVID-19 vaccination** |  |  |  |  |
| No | Ref. |  |  | Ref. |
| 1 dose | -0.675 (-5.89; 4.54) |  |  | -0.7678 (-4.67; 3.13) |
| 2 doses | 2.369 (0.06; 4.68) |  |  | 0.5563 (-1.38; 2.50) |
| 3 doses | 3.78 (0.24; 7.32) |  |  | 2.1369 (-0.84; 5.12) |
| **Social health determinants** |  |  |  |  |
| **Have someone to share personal problem with** |  |  |  |  |
| No |  | Ref |  |  |
| Yes |  | -1.9047 (-4.68; 0.87) |  |  |
| Difficult to say |  | 1.9858 (-1.15; 5.12) |  |  |
| **Relationships with family members** |  |  |  |  |
| got better |  | **-4.2476 (-7.46; -1.03) *** |  | -0.5815 (-3.16; 2.00) |
| stayed the same |  | **-5.3170 (-7.88; -2.75) ***** |  | -1.2620 (-3.43; 0.91) |
| got worst |  | Ref |  | Ref |
| **Relationships with friends** |  |  |  |  |
| got better |  | 1.2918 (-2.17; 4.75) |  |  |
| stayed the same |  | Ref |  |  |
| got worst |  | 1.9118 (0.47; 4.29) |  |  |
| **Relationships with colleagues/neighbors** |  |  |  |  |
| got better |  | **-5.8381 (-10.11; -1.58) **** |  | -2.9303 (-6.29; 0.43) |
| stayed the same |  | -4.5882 (-7.05; -2.12) *** |  | -0.7424 (-2.79; 1.30) |
| got worst |  | Ref |  | Ref |
| **Relationships with locals** |  |  |  |  |
| got better |  | **-5.3386 (-9.90; -0.77) *** |  | -0.5547 (-4.25; 3.14) |
| stayed the same |  | **-4.7043 (-8.78; -0.63) *** |  | -3.4712 (-6.74; -0.21) |
| got worst |  | Ref |  | Ref |
| **Attending meetings organized for refugees** |  |  |  |  |
| No |  | Ref |  |  |
| No, but plan to |  | -0.0621 (-2.69; 2.57) |  |  |
| Yes, but rarely |  | -0.6233 (-2.81; 1.56) |  |  |
| Yes, often |  | -1.9919 (-5.01; 1.022) |  |  |
| **Participating in Ukrainian religious communities** |  |  |  |  |
| No |  | Ref |  |  |
| No, but plan to |  | 0.8466 (-2.50; 4.19) |  |  |
| Yes |  | -0.5834 (-3.69; 2.53) |  |  |
| **Cultural differences** |  |  |  |  |
| Yes |  | 2.7719 (-0.03; 5.58) |  |  |
| No |  | Ref |  |  |
| **Discrimination** |  |  |  |  |
| Yes |  | **4.6632 (1.72; 7.61) **** |  | 1.0091 (-1.40; 3.42) |
| No |  | Ref |  | Ref |
| **Mental health determinants:** |  |  |  |  |
| **Self-Reported Emotional and Psychological Status (SREPS)** |  |  | **4.4872 (2.95; 6.021) ***** | **1.5200 (0.30; 2.75) *** |
| **Coping strategies** |  |  |  |  |
| Problem Focused Strategy (>2.5) |  |  | -0.0530 (-2.34; 2.23) | 0.0665 (-1.65; 1.78) |
| Emotion Focused Strategy (>2.5) |  |  | **4.1085 (1.702; 6.515) ***** | 1.1935 (-0.59; 2.98) |
| Avoidant Strategy (>2.5) |  |  | **5.5259 (3.06; 7.99) ***** | 1.1753 (-0.81; 3.16) |
| **Total PSS** |  |  |  |  |
| Low |  |  | Ref | Ref |
| Moderate |  |  | **6.6715 (1.30; 12.04) *** | 2.2097 (-1.49; 5.91) |
| High |  |  | **10.4988 (4.43; 16.57) ***** | 3.2506 (-1.15; 7.65) |
| **Necessary Phycological Aid** |  |  |  |  |
| Not enough |  |  | 1.6614 (-0.80; 4.12) |  |
| Enough |  |  | Ref |  |
| Difficult to say/n/a |  |  | -1.5979 (-4.33; 1.13) |  |
| **Total PHQ_9** |  |  |  | **1.0261 (0.86; 1.19) ***** |
